# Increased Association of Deamidated αA-_N101D_ with Lens membrane of transgenic αA_N101D_ vs. wild type αA mice: potential effects on intracellular ionic imbalance and membrane disorganization

**DOI:** 10.1186/s12886-020-01734-0

**Published:** 2020-12-10

**Authors:** Om Srivastava, Kiran Srivastava, Roy Joseph, Landon Wilson

**Affiliations:** 1 Department of Optometry and Vision Science, University of Alabama at Birmingham, 1716, University Boulevard, Birmingham, AL 35294-0010 USA; 2 Targeted Metabolomics and Proteomics Laboratory (TMPL), Department of Pharmacology and Toxicology, University of Alabama at Birmingham, Birmingham, AL 35294-0010 USA

**Keywords:** Lens, Crystallins, Deamidation, Post-translational modifications, Transgenic mice, Cataract

## Abstract

**Abstract:**

We have generated two mouse models, in one by inserting the human lens αAN101D transgene in CRYαA_N101D_ mice, and in the other by inserting human wild-type αA-transgene in CRYαA_WT_ mice. The CRYαA_N101D_ mice developed cortical cataract at about 7-months of age relative to CRYαA_WT_ mice. The objective of the study was to determine the following relative changes in the lenses of CRYαA_N101D_- vs. CRYαA_WT_ mice: age-related changes with specific emphasis on protein insolubilization, relative membrane-association of αA_N101D_ vs. WTαA proteins, and changes in intracellular ionic imbalance and membrane organization.

**Methods:**

Lenses of varying ages from CRYαA_WT_ and CRYαA_N101D_ mice were compared for an age-related protein insolubilization. The relative lens membrane-association of the αAN101D- and WTαA proteins in the two types of mice was determined by immunohistochemical-, immunogold-labeling-, and western blot analyses. The relative levels of membrane-binding of recombinant αA_N101D_- and WTαA proteins was determined by an in vitro assay, and the levels of intracellular Ca^2+^ uptake and Na, K-ATPase mRNA were determined in the cultured epithelial cells from lenses of the two types of mice.

**Results:**

Compared to the lenses of CRYαA_WT_, the lenses of CRYαA_N101D_ mice exhibited: (A) An increase in age-related protein insolubilization beginning at about 4-months of age. (B) A greater lens membrane-association of αAN101D- relative to WTαA protein during immunogold-labeling- and western blot analyses, including relatively a greater membrane swelling in the CRYαA_N101D_ lenses. (C) During in vitro assay, the greater levels of binding αAN101D- relative to WTαA protein to membranes was observed. (D) The 75% lower level of Na, K-ATPase mRNA but 1.5X greater Ca^2+^ uptake were observed in cultured lens epithelial cells of CRYαA_N101D-_ than those of CRYαA_WT_ mice.

**Conclusions:**

The results show that an increased lens membrane association of αA_N101D_-_−_relative WTαA protein in CRYαA_N101D_ mice than CRYαA_WT_ mice occurs, which causes intracellular ionic imbalance, and in turn, membrane swelling that potentially leads to cortical opacity.

## Background

Although the cornea is the primary refractive tissue performing 70–80% of refraction of the eye, the major function of the lens is in accommodation and to partly help in the refraction. The lens accommodative function gradually diminishes with age, and is almost completely lost at age of > 50 years. The lens transparency plays an important role in focusing light on to the retina, but this role is gradually lost as it develops age-related opacity. Several unique factors maintain lens transparency for up to > 60 year of our life time. These include: cellular homeostasis among only two types of cells (epithelial and fiber cells) [1], an orderly terminal differentiation of epithelial to fiber cells with precise organelles loss [2], the unique interactions among crystallins [3], with almost no protein turnover [4], the specialized lens metabolism [5], specific interactions among α-crystallin and membrane [6], the precise maintenance of intracellular and extracellular ionic concentrations [7], the low levels of cellular water and oxygen in the lens inner cortex and nuclear regions [8], and a unique membrane lipid composition [9]. Alterations among some of these lens unique factors play direct or indirect roles in pathogenesis of cataracts (e.g., pediatric- and age-related cataracts). However, additional cataract-causative factors are also identified, which include mutations in crystallins [10], oxidative insults of crystallins, the loss of redox balance of glutathione [11], extensive truncations of α-, β-, and γ-crystallins [12–20], a variety of post-translational modifications with deamidation as being the most abundant [21–25], and the loss of membrane integrity [7, 26, 27]. These factors individually or in combination also cause lens opacity through altered lens cellular structures and contents, ionic imbalance, increased water and oxygen levels, loss of natural interactions among crystallins, and crystallins’ unfolding, degradation and cross-linking.

Our focus in this study is the potential roles of deamidation of Asn_101_ of αA crystallin to Asp that introduces negative charges and shown to alter their hydrophobicity, tertiary structures, crystallin-crystallin interactions, and leads to aggregation and cross-linking [21–27]. In this study, the deamidation of Asn_101_ to Asp in in a mouse model was studied to determine phenotypic and molecular changes within the lens due to deamidation of a single nucleotide change in CRYAA crystallin gene. This site was chosen because our past study showed that only deamidation of Asn localized at specific sites in crystallins (e.g., deamidation of N101 but not of N123 residues in αA-crystallin [24], and of N146 but not of N78 of αB-crystallin) exhibited the above-described deamidation-induced effects [25]. To show the potential effects of deamidation in vivo, we have generated mouse models by inserting the human lens αA-N101D transgene in CRYαA_N101D_ mice, and human lens wild-type αA-transgene in CRYαA_WT_ mice (to act as a control). The CRYαA_N101D_ mice developed cortical cataract at about 7-months of age relative to CRYαA_WT_ mice [28, 29]. This model showed for the first time that in vivo *expression* of the deamidated αAN101D caused cortical lens opacity, which was due to the disruption of fiber cell structural integrity and protein insolubilization as aggregation [28]. The comparative RNA sequencing and Ingenuity Pathway Analyses (IPA) of lenses from 2- and 4-months old CRYαA_N101D_- and CRYαA_WT_ mice showed that the genes belonging to cellular assembly and organization, cell cycle and apoptosis networks were altered in αA_N101D_ lenses [29]. This was accompanied with several cellular defects in αA_N101D_ lenses that included defective terminal differentiation (increased proliferation and decreased differentiation) of epithelial cells to fiber cells, and reduced fiber cells denucleation and expressions of Rho A and Na, K-ATPase (the major lens membrane-bound molecular transporter) [29]. The findings also suggested the potential role of lens intracellular ionic imbalance as the major reason for the development of cataract [29]. The above findings suggested that the altered intracellular ionic imbalance could be due to potential loss of membrane integrity that caused cortical opacity at about 7-months of age in the CRYαA_N101D_ mouse model. Therefore, the focus of the present study was to determine whether an increased membrane-association of αA_N101D_ potentially compromises membrane integrity, and causes an ionic imbalance and leads to cataract development.

## Methods

### Ethics statement

All animal experiments were performed per protocols approved by the Institutional Animal Care and Use Committee (IACUC) of the University of Alabama at Birmingham (Protocol no. 130208393). Mice were housed in a pathogen-free environment at the facility of the University of Alabama at Birmingham.

### Materials

Unless stated otherwise, the molecular biology-grade chemicals were purchased from Millipore-Sigma (St. Louis, MO, USA) or Fisher (Atlanta, GA, USA) companies. The Rabbit polyclonal anti-human aquaporin-0 (AQP0) antibody was purchased from Alpha Diagnostics (San Antonio, TX, USA). Additional commercial sources of various chemicals and antibodies used in the study are described throughout the text.

### Generation of transgenic mice

The mouse model that expresses a human αA-crystallin gene in which Asn-101 was replaced with Asp is referred to as αA_N101D_-transgenic mouse model. This model has been considered to be “deamidated” in this study, and the mice expressing αAN101D-transgene is referred here as CRYαA_N101D_ mice. Both mouse models (human lens αA_N101D_ transgenic- and human wild-type αA-transgenic mouse models were generated in Dr. Om Srivastava’s laboratory [28]. Independent transgenic (Tg) mouse lines were established from transgenic founders using C57BL/6 mice (Harlan Laboratories, Indianapolis, IN). αAN101D protein expression constituted about 14 and 14.2% of the total αA in the lens WS-and WI-proteins of the αA_N101D_ transgenic mice, respectively [28]. The mouse lenses were extracted after the mice were euthanized using the CO_2_ procedure as per approved method by the Institutional Animal Care and Use Committee (IACUC) of the University of Alabama at Birmingham (Protocol no.130208393). Adult (2–3 months) wild type mice (C57BL6) were obtained from the university breeding colony. Animals were kept under a 12/12 h light–dark cycle and had ad libitum access to food and water. We have used three mice from each group of CRYαA_WT_ mice control and αA_N101D_ mice in all the experiments described below.

### Isolation of water soluble (WS)- and water insoluble (WI)-proteins from mouse lenses

The WS- and WI-protein fractions from lenses of desired ages of CRYαA_WT_- and CRYαA_N101D_ mice were prepared as previously described by us [28]. All procedures were performed at 5 °C unless specified otherwise. The lenses were removed under a dissecting microscope and placed in 5 °C-cold buffer A (5 mM Tris-HCl, 1 mM EDTA, 1 mM DTT, pH 7.8, and protease inhibitor cocktail [Roche Life Science, Indianapolis, IN]), and centrifuged at 14,000 x g for 15 min at 5 °C to separate the WS- and WI- protein fractions. The supernatant (WS-protein fraction) was collected, and next the pellet (WI-protein fraction) was resuspended in buffer A, centrifuged as above. The recovery of WS- and WI-protein fractions was repeated twice after centrifugation, and the WS supernatants after each centrifugation steps were pooled. The final WI-protein pellet was solubilized in 5 mM Tris-HCl, pH 7.5, containing 8 M urea, 5 mM EDTA, and 5 mM EGTA. The 8 M urea concentration was diluted to 4 M urea with buffer A prior to centrifugation as above. The protein concentrations in these fractions were determined by using a kit (Pierce Biotechnology-Thermo Fisher) using bovine serum albumin as a standard.

### Membrane isolation from mouse lenses

The membranes from lenses of 1- and 6-month-old CRYαA_WT_ and CRYαA_N101D_ mice were prepared as described previously [30, 31]. Lenses of identical ages from both types of mice were homogenized in buffer B (0.05 M Tris-HCl, pH 8.0 containing 5 mM EDTA, 1 mM DTT, 150 mM NaCl, and protease inhibitor cocktail [Roche, Indianapolis, IN]), and the preparations were centrifuged at 100,000 x g for 30 min using Beckman TL 100 centrifuge with a TLA 100.3 rotor. The supernatant was collected, and the pellets were washed twice with the above buffer B and centrifuged as above. This was followed by three additional washes with buffer B containing 8 M urea and centrifugation as above after each wash. Next, the pellet was washed twice with water and centrifuged as above. The pellet was then washed with 0.1 M cold (5 °C) NaOH [30, 31]. A final wash of pellet was with water and centrifugation as above to recover the purified lens membrane preparations as pellets.

### Purification of recombinant WTαA- and αA-_N101D_-crystallins, their conjugation with Alexa Fluor 350 and membrane binding

The WTαA- and αA-N101D mutant proteins were expressed in *E.coli* and purified by a Ni-affinity column chromatographic method as previously described by us [28]. Each protein was labeled with Alexa-350 using a protein labeling kit as suggested by the manufacturer (Molecular Probes, Carlsbad, CA). The binding of Alexa 350-conjugated WT αA- and αA-N101D mutant proteins to mouse lens membrane (isolated from C57BL non-transgenic mice) was determined as previously described [32, 33]. During the binding assay, the purified lens membrane (containing 2.5 mg protein; isolated from 1 to 3-month old non-transgenic C57 mice) was incubated with increasing but identical concentrations of either Alexa-labelled WT αA- or αA-_N101D_ proteins at 37^ο^C for 6 h. Next, the incubated preparations were centrifuged at 14,000 X g and the supernatant and pellet (membrane fraction) recovered. After washing the membrane fraction with water and centrifugation as above, the relative levels of fluorescence of membranes incubated with WT αA- and αA-_N101D_ mutant proteins were determined using Perkin Elmer Multiplate Reader (Model Victor1420–04).

### Determination of intracellular Ca^2+^ in epithelial cells in culture from lenses of CRYαA_WT_ - and CRYαA_N101D_ mice

To culture epithelial cells, six 5-months old lenses from CRYαA_WT_ - and CRYαA_N101D_ mice were excised and incubated with 0.25% trypsin at 37^ο^ C for 2.5 h in an incubator with 5% CO_2_-humidified air. Next, the lens cells in trypsin solution were centrifuged at 1200 rpm for 3 min, and trypsin (in the supernatant) was discarded. The lens epithelial cells (recovered as pellet) were suspended in medium 199 (Thermo Fisher Scientific, Grand Island, NY) containing 10% fetal calf serum (Hyclone, Logan, Utah) and 1% antibiotic-antimycotic solution (Thermo Fisher Scientific, Grand Island, NY) in 12-well plates (Corning, Franklin Lakes, NJ). After 24 h, the unattached cells were discarded by washing with the above medium. The old medium was replaced with fresh medium after every 48 h, and the cells were allowed to grow for 7 to 10 days until confluent. Next, the confluent cells were trypsinized and seeded in 12-well plates for intercellular Ca^2+^ determination and were allowed to grow for 24 h. The cells were washed with medium 199 without phenol red, incubated in calcium orange dye (Thermo Fisher Scientific, Grand Island, NY) at a final concentration of 4 μM for 30 min at room temperature as instructed in the manufacturer’s protocol. After 30 min, the cells were washed with the above medium, and Ca^2+^ indicator was examined under a microscope (Leica DMI 4000B) using a Texas Red filter.

### Western blot and Immunohistochemical analyses

The WS- and WI-proteins and membrane fractions isolated from lenses were analyzed for their immunoreactivity with anti-aquaporin-0 antibody (to visualize the membrane intrinsic protein), and Mouse anti-His monoclonal antibody ([Novagen, Madison, WI], to visualize WTαA and αA_N101D_) during Western blot analysis. The SDS-PAGE analysis was carried out as described by Laemmli [34].

The confocal immunohistochemical analysis of lens axial sections of WTαA and αA_N101D_ was carried out as previously described by us [28]. The analysis was performed at the High-Resolution Imaging core facility of the University of Alabama at Birmingham.

### Localization by Immunohistochemical-transmission Electron microscopic method

The analysis was performed at the High-Resolution Imaging core facility of the University of Alabama at Birmingham. His-tagged αA_WT_- and αA_N101D_-crystallins were localized in lens cells by an Aurion immunogold method and the reagents used were Aurion Conventional Immunogold reagents (Electron Microscopy Science [PA]). Lenses of desired ages were fixed in phosphate-buffered saline, pH 7.4 containing 4% paraformaldehyde and 0.05% glutaraldehyde (Electron Microscopy Sciences, Hatfield, PA) for 2 h at room temperature, and then overnight at 4 °C. The fixed lenses were washed with water (Millipore, Billerica, MA). Samples were dehydrated by ascending ethanol gradient series followed by infiltration overnight at 4 °C with absolute ethanol: London Resin (LR) white (1:1). Next, the samples were incubated overnight with pure LR white resin on a rotating platform. The lenses were removed and transferred to gelatin capsules containing fresh LR white and allowed to polymerize for 24 h at 45–50 °C. Ultra-thin (silver gold to light gold) LR white lens sections were collected on nickel mesh grids. The color of sections was silver-gold to light gold, and based on their color, the thickness was estimated to between 70 and 80 nm. For immunogold-labeling, the protocol as described in Electron Microscopy Sciences (Hatfield, PA) was precisely followed. To inactivate aldehyde groups present after aldehyde fixation, the samples on grids were incubated on 0.05 M glycine in PBS buffer for 10–20 min. Next, the grids were transferred onto drops of the matching Aurion blocking solution for 15 min, and then were washed for 15 min in incubation solution (PBS containing 0.1% bovine serum albumin and 15 mM NaN3, pH 7.3). This was followed by a 2X wash in incubation buffer, each time for 5 min. The grids were incubated with two primary antibodies (Mouse anti-His monoclonal antibody and Rabbit anti-aquaporin-0 polyclonal antibody for 1 h. In controls, the primary antibodies were omitted. The grids were then washed 6X (5 min each time) with the incubation solution and transferred to following secondary antibody conjugates {(goat anti-Rabbit EM grade conjugate 25 nm diameter) and (goat anti- mouse EM grade conjugate 10 nm diameter)} and were incubated for 30 min to 2 h. The grids were washed on drops of incubation solution for 6X (5 min each time). The grids were washed twice with PBS for 5 min, post-fixed in 2% glutaraldehyde in PBS for 5 min, and finally washed with distilled water and contrasted according to standard procedures. Lens sections were imaged using an FEI 120kv Spirit TEM (FEI-Thermo Fisher), and images were collected using an AMT (AMT-Woburn, MA) digital camera.

### RNA extraction and reverse transcription-quantitative polymerase chain reaction (RT-PCR [qPCR])

RNA was extracted with Trizol reagent (Invitrogen) from cultured lens epithelial cells from CRYαA_N101D_ and CRYαA_WT_ mice, and all the samples were analyzed in triplicates. Real-time PCR quantifications were performed using the BIO-RAD iCycler iQ system (Bio-Rad, Hercules, CA), using a 96-well reaction plate for a total volume of 25 μL. RNA was extracted as described above. Primers were designed using Primer3 for the following genes:

**Atp1a2** Forward-5’CGGGAGCCATAAGGGTTTGT 3′, and **Atp1a2** Reverse- 5’GCACTGACTTGGCTGTTGTG 3′.

The ACTB gene was used for normalization. The reaction mixture included 12.5 μL of Real- Time SYBR Green PCR master mix, 2.5 μL of reverse transcription product, 1 μL of forward and reverse primer and 8 μL of DNase/RNase free water. The reaction mixtures were initially heated to 95 °C for 10 min to activate the polymerase, followed by 40 cycles, which consisted of a denaturation step at 95 °C for 15 s, annealing at 55 °C for 60 s and an elongation step at 72 °C. The qRT-PCR data were analyzed by the comparative ΔCt method.

## Results

### Age-related protein Insolubilization in lenses of CRYαA_N101D_ and CRYαA_WT_ mice

**Fig. 1:**
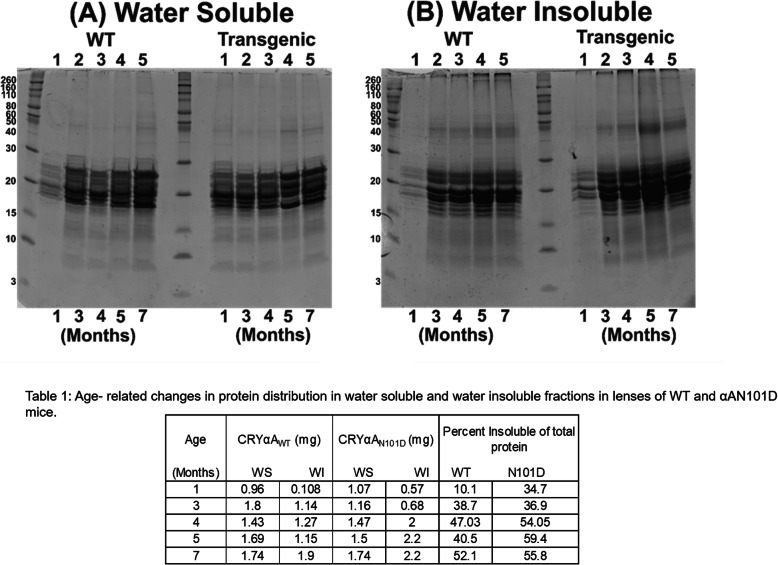
SDS-PAGE analysis of WS- and WI-proteins from lenses of CRYαA_WT_- and CRYαA_N101D_ mice at different ages. **a**. Coomassie blue stained gel of WS protein from both WT and Transgenic mice at different ages as indicated at the bottom of the gel (months). And the numbers 1–5 at the top of the gel indicate lanes. **b** Coomassie blue stained gel of WI protein from both WT and Transgenic mice at different ages as indicated at the bottom of the gel (months).). Note that a greater insolubilization and aggregated proteins (M_r_ > 30 kDa) were seen in WI-proteins of lenses of 4-month and older CRYαA_N101D_mice compared to age-matched lenses from CRYαA_WT_ mice. The Table 1. shows quantification of protein levels in the WS- and WI-protein fractions of lenses of different ages from CRYαA_N101D_ and CRYαA_WT_ mice. Gel images are not cropped

**Fig. 2:**
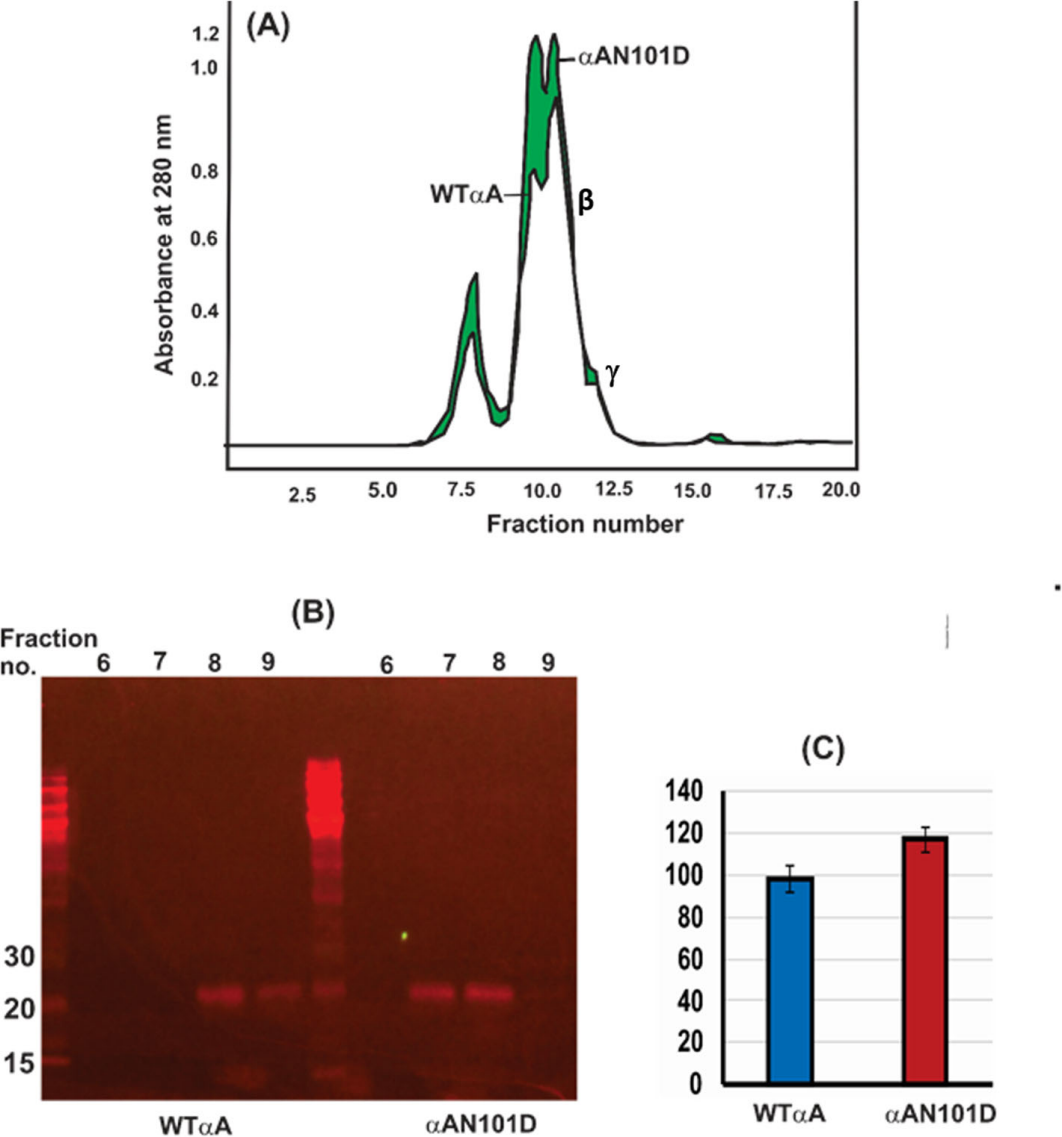
Size-exclusion HPLC (using a G-4000PWXL column) and SDS-PAGE analysis of WS-HMW proteins eluted in the void volume. **a** HPLC-protein elution profiles at 280 nm of WS-proteins from lenses of 5-month-old CRYαA_N101D_ - and CRYαA_WT_ mice. The green region shows the difference in the A280 profiles of WS-proteins from the two type of lenses. **b** Western blot analysis of the void volume peaks (constituted by the fraction no. 6 to 9 in [A]) following HPLC separation of WS-proteins from lenses of CRYαA_N101D_ - and CRYαA_WT_ mice. Note that in the CRYαA_WT_ lenses, the αA-immunoreactive bands were in fractions no. 8 and 9 whereas it were in fraction no. 7 and 8 in CRYαA_N101D_ lenses, suggesting a higher M_r_ of HMW proteins in the latter. **c** Quantification of the Western Blot using Image J. Gel images are not cropped

To determine at what age there is change in the protein profiles in lenses of CRYαA_N101D_ and CRYαA_WT_ mice occurred, a comparative analysis of WS-proteins and WI-proteins from the lenses of the two types of mice of different ages was carried out *(*Fig. 1). The WS- and WI-proteins from lenses of different ages (1-, 3-, 4-, 5- and 7-months) were analyzed by SDS-PAGE. The WS-protein profiles from the lenses of the CRYαA_N101D_ and CRYαA_WT_ mice were almost identical until 3-months of age, except lens preparations from ages of 4-, 5- and 7-months of CRYαA_N101D_ mice exhibited relatively greater levels of aggregated protein of M_r_ > 30 kDa and higher relative to same-aged lenses from CRYαA_WT_ mice (Lanes 4 and 5 in Fig. 1b). Additionally, on quantification, the relatively increasing levels of WS-proteins showed age-related water insolubilization beginning at 4-months of age in the lenses of αA_N101D_ mice (Table 1). Between 4- to 7-months of age, relatively about 5 to 10% higher proteins became insoluble in lenses of CRYαA_N101D._To determine changes in individual crystallins due to their insolubilization, the WS-protein fraction from 7-month-old lenses was fractionated by a size-exclusion HPLC using a G-4000PWXL column (Tosoh Biosciences, fractionation range of protein with M_r_’s between 1X10^4^ to 1X10^7^ Da). The comparative protein elution profiles at 280 nm of 7-month old lenses of αA_N101D_-mice showed an increased protein in the void volume peak (representing WS-HMW proteins), and reduced β- and γ-crystallin peaks relative to lenses of CRYαA_WT_-mice (the differences shown in green in Fig. 2a). The void volume peak in WS-protein fraction was also higher in the 7-month old lenses relative to 1-month old lenses of αA_N101D_-mice (Results not shown), suggesting a relatively increased HMW protein aggregate formation with aging. On western blot analysis of the individual column fractions nos. 6 to 9 (constituting the void volume-HMW-protein peak) with an anti-His antibody, the levels of His-immunoreactive protein were higher in 7-month old CRYαA_N101D_ lenses compared to the identical aged CRYαA_WT_ lenses (Fig. 2b). Additionally, because the immunoreactive peak in the WT lenses was in the fractions no. 8 and 9 whereas it was in the fractions no. 7 and 8 in the αA_N101D_ lenses that suggested that the HMW proteins of αA_N101D_ lenses showed a higher molecular weights relative to the HMW proteins from WT lenses. On quantification of Western blot images with Image J (Fig. 2c), the intensity of the immunoreactive HMW proteins of αA_N101D_ was about 20% greater relative to WT lenses. Together, the results suggested a greater aggregation with higher M_r_ of the HMW-proteins in CRYαA_N101D_ lenses compared to the identical aged CRYαA_WT_ lenses.

**Table 1:** Age-related changes in Protein distribution in water soluble and water insoluble fractions in lenses of WT and αA-_N101D_ mice

Age (Months)	CRYαA_WT_ (mg)		CRYαA_N101D_ (mg)		Percent Insoluble of total Protein	
	WS	WI	WS	WI	αA_WT_	αA_N101D_
1	0.96	0.108	1.07	0.57	10.1	34.7
3	1.8	1.14	1.16	0.68	38.7	36.9
4	1.43	1.27	1.47	2.0	47.03	54.05
5	1.69	1.15	1.5	2.2	40.5	59.4
7	1.74	1.9	1.74	2.2	52.1	55.8

### Identification proteins present in water insoluble-urea soluble (WI-US) - and water insoluble urea insoluble (WI-UI) protein fractions of lenses of CRYαA_WT_ and CRYαA_N101D_ mice

**Fig. 3:**
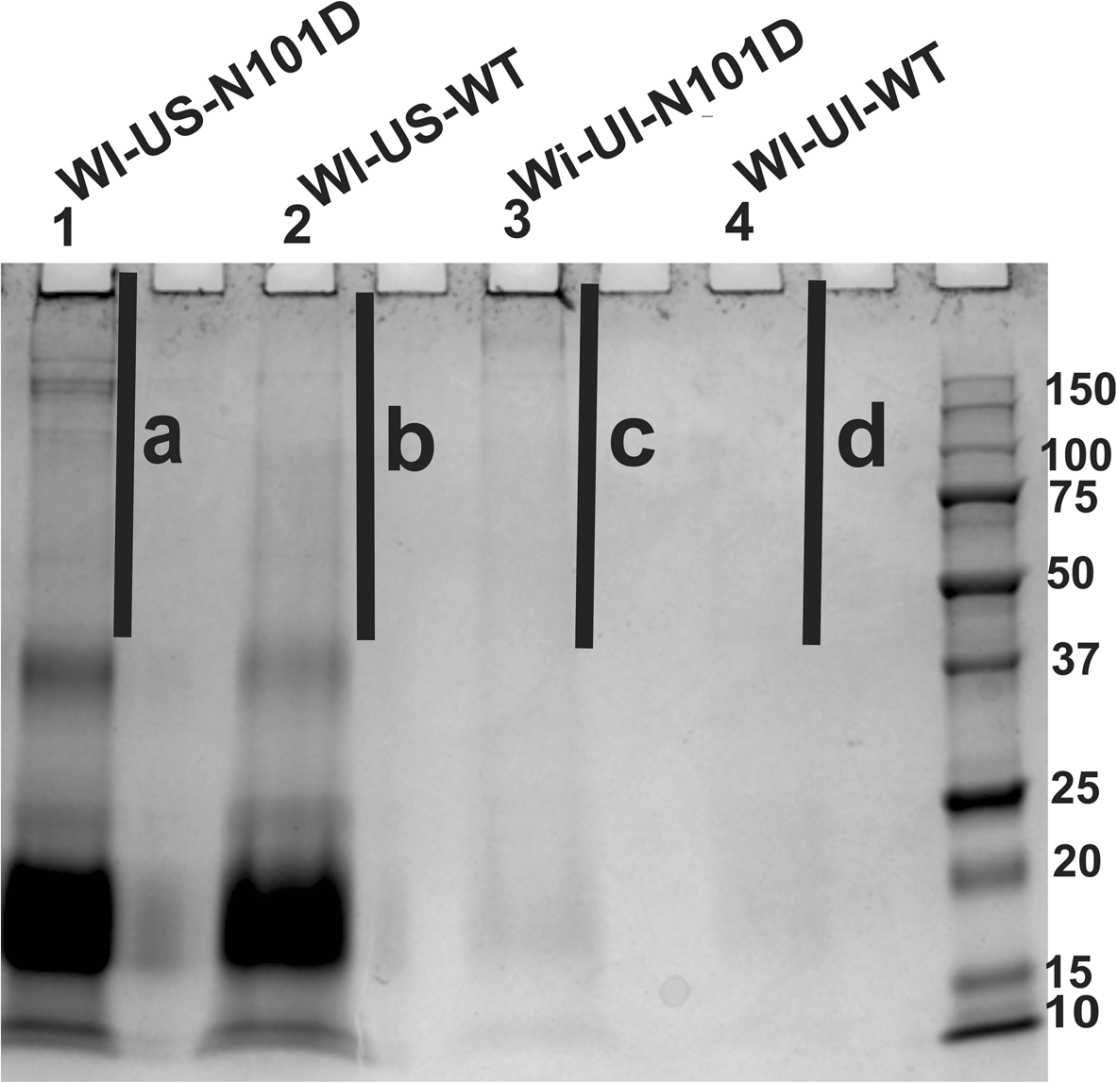
SDS-PAGE analysis of WI-US- and WI-UI-protein fractions of 5-month-old lenses from CRYαA_N101D_ and CRYαA_WT_ mice. To normalize the protein analyses, the protein fractions were isolated under identical conditions and with identical buffer volumes. Equal volumes of protein fractions from lenses of two types of mice were used during the analysis. The four fractions numbers as 1 to 4 (containing total proteins in WI-US- and WI-UI-fractions) and four fractions numbered as a to d (containing aggregated proteins with Mr. > 30 kDa) were analyzed by mass-spectrometric methods, and the results are shown in Supplementary Tables A, B, C and D. Gel images are not cropped

To Identify the insolubilized proteins in WTαA vs. αA_N101D_ lenses, the WI-proteins from 5-month-old mice were further fractionated into WI-US- and WI-UI-protein fractions, and examined by SDS-PAGE (Fig. 3) followed by their protein compositional analysis by mass spectrometry. SDS-PAGE analysis showed that both WI-US- and WI-UI-protein fractions from CRYαA_N101D_ lenses contained greater levels of protein species including aggregated proteins (M_r_ > 30 kDa) [Identified as a, and c in Fig. 3] relative to the same fractions from lenses of CRYαA_WT_ mice (Identified as b and d in Fig. 3). The mass spectrometric analysis was carried out at the following two levels: (i) In the first level analysis, determination of the total protein compositions in the WI-US- and WI-UI protein fractions of the two types of lenses (Supplemental Tables A [Comparative protein compositions of WI-US-fractions of CRYαA_N101D_ and WTαA lenses], and Supplemental Table B [Comparative protein compositions of WI-UI-fractions of αA_N101D_ and WTαA lenses]). (ii) In the second level analysis, the protein compositions of protein aggregates (M_r_ > 30 kDa) in WI-US-fraction of αAN101D lenses (Identified as ‘a’ in Fig. 3), and WI-US-protein fraction of WTαA lenses (Identified as ‘b’ in Fig. 3) [Supplemental Table C]. Similarly, the compositions of protein aggregates (M_r_ > 30 kDa) in WI-UI-fraction of αA_N101D_ lenses (Identified as ‘c’ in Fig. 3,), and WI-US-fraction of WTαA lenses (Identified as ‘d’ in Fig. 3) were determined [Supplemental Table D]. The rationale of the two levels of analysis was to determine the relative proteins compositions due to the greater insolublization of proteins in CRYαA_N101D_ lenses relative to CRYAAWT lenses (Fig. 1, Table 1). Our expectation was that the level 1 comparative examination would identify the total proteins that underwent insolubilization, and existed in the US- and UI-protein fractions, whereas the level 2 analysis would selectively identify those proteins that formed aggregates (M_r_ > 30 kDa) in the US- and UI-fractions. The rationale was that the information would implicate potential roles of specific crystallins in the aggregation and therefore, in the cataractogenic mechanism.

(i). **Comparative Protein Compositions in WI-US Fractions of Lenses from CRYαA**_**N101D**_ **and CRYαA**_**WT**_ **Mice**

The proteins detected in the WI-US-protein fractions of CRYαA_N101D_ lenses but were absent in the *WT* lenses are described in Supplemental Table A. Together, the results show that the WI-US fraction of CRYαA_N101D_ lenses was enriched in several histones, which could be due to the lack of denucleation relative to *WT* lenses. Absence of Retinal dehydrogenase in transgenic lens fraction.

(ii). **Comparative Protein Compositions of WI-UI-Fractions of Lenses from CRYαA**_**N101D**_ **and CRYαA**_**WT**_ **Mice**

The proteins present in the WI-UI-protein fractions of CRYαA_N101D_ lenses but were absent in WT lenses are described in Supplemental Table B. In summary, the results again show that the majority of histones that existed in CRYαA_N101D_ lenses were absent in the WT lenses, which could be due to the lack of denucleation in the lenses of former mice. Also, specifically αB- and βB2-crystallin became insoluble as their levels were higher even in the WI-UI-fraction of lenses of CRYαA_N101D_ relative to WT lenses.

(iii). **Compositions of Aggregated Proteins (M**_**r**_ **> 30 kDa) in WI-US- and WI-UI-Fractions of Lenses from CRYαA**_**N101D**_ **and CRYαA**_**WT**_ **Mice**

As noted above, the purpose of the second level of mass spectrometric analysis was to elucidate the comparative compositions of aggregated proteins (M_r_ > 30 kDa) in WI-US- and WI-UI-protein fractions of CRYαA_N101D_ and CRYαA_WT_ lenses [Supplemental Tables C and D]. On comparison, the major proteins present as aggregates (M_r_ > 30 kDa) in WI-US fraction of CRYαA_N101D_ but absent in CRYαA_WT_ were (Supplemental Table C): βB3- and γC-crystallins, collagen alpha-1(IV) chain and -alpha-2(IV) chain and nestin. In contrast, the exclusively present major proteins in WI-US fraction of CRYαA_WT_ were: γC-, γD-, γE- γF-crystallins. The above list describes the selective proteins that were water insoluble-urea soluble and became the part of the complexes with M_r_ > 30 kDa in CRYαA_N101D_ lenses. The greater abundance of αA-, and βB1-crystallins in the aggregated form suggested their potential involvement in the aggregation process along with βB3- and γC-crystallins.

On comparison of major proteins that existed in WI-UI protein fraction as > 30 kDa aggregates in CRYαA_N101D_ not in the CRYαA_WT_ included [Supplemental Table D]: γB-, γD- and γE-crystallins, and nestin. In the WI-UI fraction, the greater abundance of proteins in CRYαA_N101D_ compared to CRYαA_WT_ were: αA-crystallin and lens fiber intrinsic proteins. Together, the results showed that the proteins that remained urea insoluble and were possibly associated with the membrane of CRYαA_N101D_ lenses included: γB-, γD- and γE-crystallins, and nestin (Nestin is an intermediate filament protein).

### Increased association of αAN101D with Lens membrane in the outer cortical Fiber cells relative WTαA in CRYYAAWT lenses

Our previous report [28] showed an increased levels and abnormal deposition of αA_N101D_ within the outer cortical region in CRYαA_N101D_ lenses compared CRYαA_WT_ lenses. This suggested a relatively greater membrane binding of αA_N101D,_ which was further investigated in experiments as described below.

(i). **Immunohistochemical Analyses of Lenses from CRYαA**_**N101D**_ **and CRYαA**_**WT**_ **Mice**

**Fig. 4:**
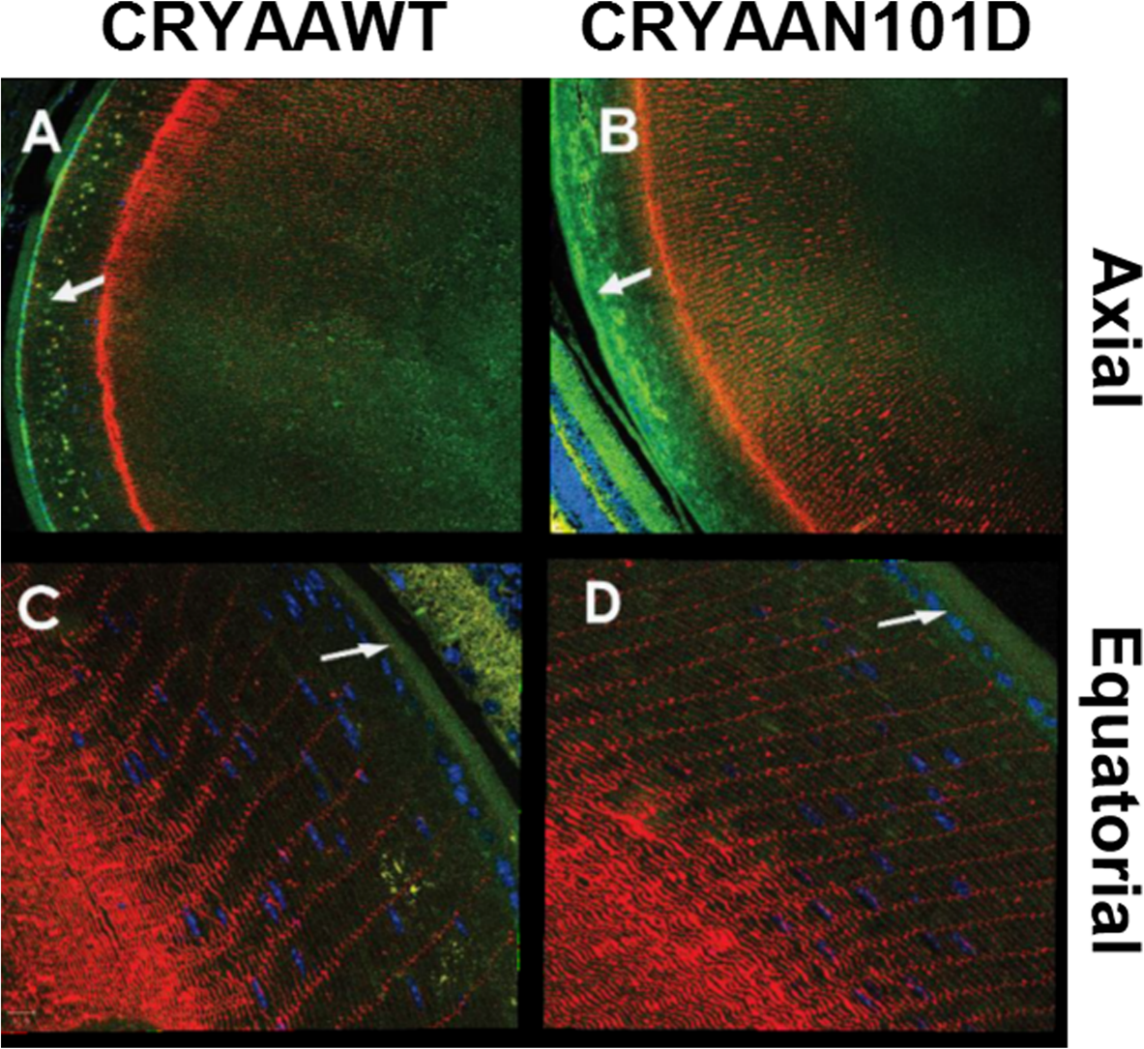
**a** Confocal-immunohistochemical analysis of 5-month old lenses from CRYαA_N101D_ and CRYαA_WT_ mice by using anti-His monoclonal (green, for αA detection)- and polyclonal anti-aquaporin 0 (red, for membrane detection)-antibodies. **a** and **b**: The axial sections at 10X magnification showed an irregular deposition of His-tagged αA (Green) in the lens outer cortex of CRYαA_N101D_ mice (in B, shown by an arrow) relative to CRYαA_WT mice_ (in A, shown by an arrow). The equatorial sections (at 40X magnification) show a greater deposit of green fluorescence in the outer cortex of CRYαA_N101D_ lens relative to CRYαA_WT_ (shown by arrows in **c** and **d**)

The purpose of the experiments was to determine relative levels of αAN101D and WTαA in the outer cortical regions of CRYαA_N101D_- vs. CRYαA_WT_ lenses. This was examined by immunohistochemical analysis of 5-months old lenses of the two types of mice using anti-His monoclonal (for detection of WTαA and αA_N101D_ [green fluorescence]) - and polyclonal anti-aquaporin 0 (for membrane detection [red fluorescence])-antibodies (Fig. 4). The axial sections (at 10X magnification) showed an irregular and greater deposition of His-tagged αA (Green) in the lens outer cortex of CRYαA_N101D_ mice (Shown by an arrow in Fig. 4a) relative to CRYαA_WT_ mice (Shown by an arrow in Fig. 4b). Similarly, the equatorial sections (at 40X magnification) also exhibited a greater immunoreactive green fluorescence in the outer cortex of the CRYαA_N101D_ lens relative to the CRYαA_WT_ lens (shown by arrows in Fig. 4c and d). Together, the results suggested the abnormally greater levels of association of αA_N101D_ in the outer cortical regions, and potentially with the fiber cell membranes in the CRYαA_N101D_ lenses relative to those of CRYαA_WT_ lenses.

(ii). **Relative Membrane-Association of WTαA- and αA**_**N101D**_ **in Lenses of CRYαA**_**N101D**_ **and CRYαA**_**WT**_ **Mice**

**Fig. 5:**
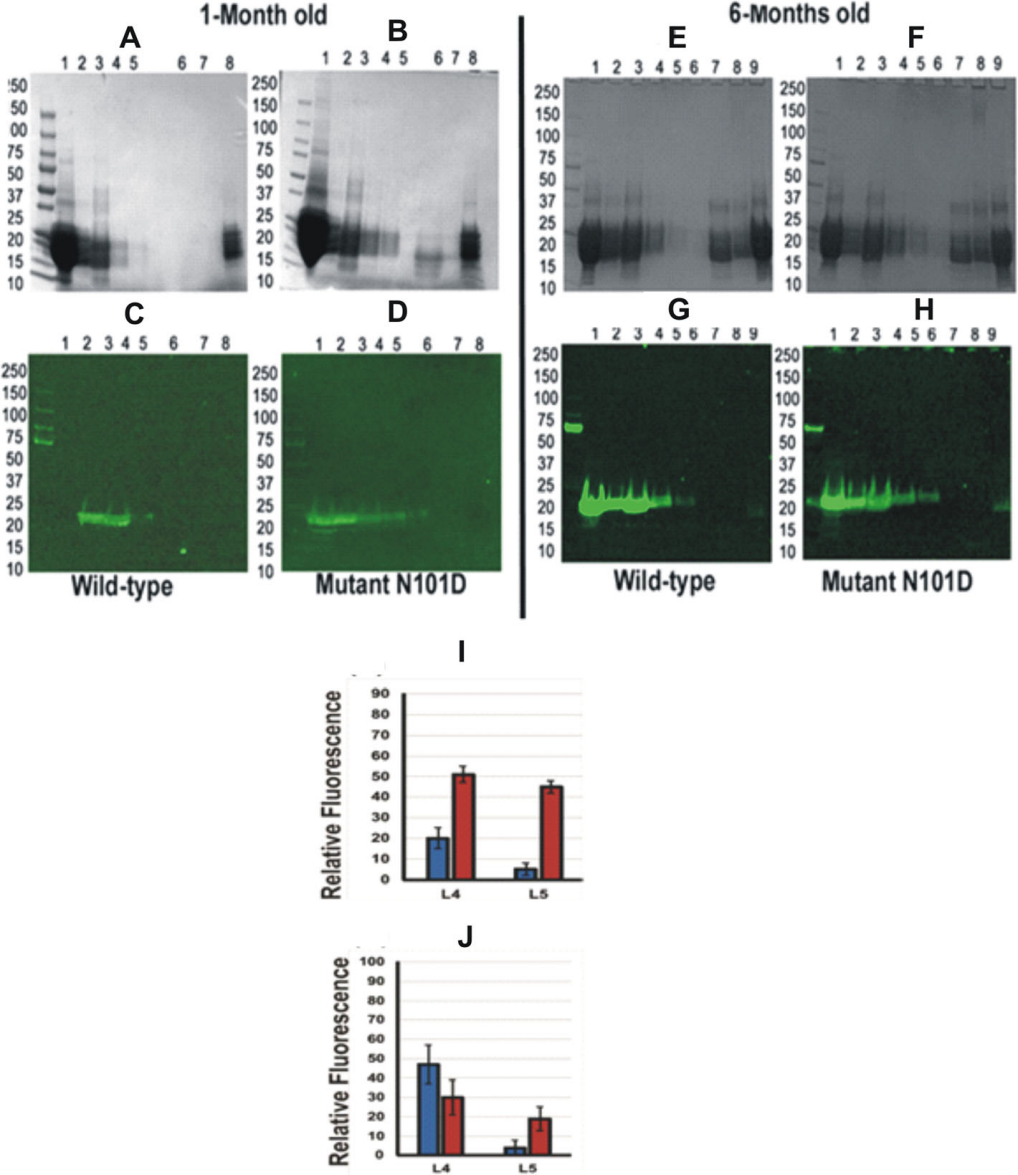
Relative Membrane-Association of WTαA- and αA_N**101D**_ in Lenses of CRYαA_N101D_ and CRYαA_WT_ Mice. **a**, **b**, **e** and **f**: the relative levels of association of WTαA and αA_N101D_ with the purified membrane preparations at different membrane purification steps analyzed using SDS-PAGE analysis at two different age groups (1 and 6 months) (**c**, **d**, **g** and **h**): Similarly the samples were analyzed by Western blot using anti-His antibody at two different age groups (1and 6 months). Additionally, in both left and right panels, the lanes 1, 2 and 3 show the WS-protein fractions recovered after 1st, 2nd and 3rd consecutive washes in buffer B to solubilize WS-proteins, respectively. Lanes 4 and 5 represent the urea soluble-protein fractions recovered during two consecutive washes of WI-protein pellet with buffer B containing 8 M urea, similarly, lanes 7 and 8 from 6-month old lenses (E and F) represent purified membrane preparation. Lane 9 of 6-month old lenses represents crude WS-homogenate. **i** & **j**: Quantification of immunoreactive bands of αA- recovered in urea-soluble fractions (Lane 4 [L4] and lane 5 [L5] represent two consecutive urea wash of WI proteins during membrane isolation from lenses of CRYαA_WT_ and CRYαA_N101D_mice as shown in Western blot analysis in Fig. 5. **i** Relative levels of immunoreactive WTαA lenses (blue) and αA-N101D αA- (red) during membrane purification from 1-month old lenses. **j** Relative levels of immunoreactive WTαA lenses (blue) and αA_N101D_ αA- (red) during membrane purification from 6-months old lenses. Note that relatively higher levels of αA_N101D_ than WTαA was associated with purified membranes in lanes 4 (in 1-month old) and lane 5 (in both 1- and 6- month-old) of the two types of lenses. Gel images are not cropped

The rationale for the next experiment was that if the greater membrane-association of αA-_N101D_ occurs in vivo in CRYαA_N101D_ lenses compared to CRYαA_WT_ lenses, the difference in their levels could also be determined in the purified membrane fractions by western blot analysis. The expectation was that following the step-wise membrane purification by using 8 M urea (to dissociate non-covalently-bound membrane proteins), and by the final wash with 0.1 N NaOH (to remove non-membranous extrinsic proteins) [30, 31], the purified membrane would show relative levels of membrane-association of αA_N101D_ vs. WTαA in the two types of mice. To normalize the levels of the relative association during the membrane preparations, two lenses of 1-month-old and two lenses from 6-month old from CRYαA_N101D_ and CRYαA_WT_ mice were identically processed, using identical volumes of buffers at each steps during membrane purification (See Methods). Next, Western blot analysis using anti-His- and anti-aquaporin 0-antibodies were used to determine the relative levels of membrane-association of WTαA and αAN101D at different purification steps (Results not shown). To simplify the western blot results of fractions recovered during the sequential steps of membrane purification, only the results of immunoblots with anti-His antibody but not with anti-aquaporin-0 are shown in Fig. 5. However, the western blot profiles with anti-aquaporin-0 were almost identical to anti-His antibody results. In Fig. 5, a, b, e and f show SDS-PAGE analysis followed by Coomassie blue-stained gels exhibiting relative levels of protein bands in preparations at different membrane purification steps in lenses at two different age groups (1 and 6 months). In Fig. 5, c and d (1-month old lenses) and G and H (6-months old lenses) corresponded to samples of A, B, E and F (Coomassie blue-stained gels), and show the Western blot results with anti-His antibody (green fluorescence) in the two different age groups (1 and 6 months). The levels of green fluorescence with His-tagged αA in lenses of 1-month old lenses (Fig. 5, left panel: WTαA [C] and αA_N101D_ [D]) and 6-month old lenses (Fig. 5, right panel: WTαA [G] and αA_N101D_ [G]) are shown. Additionally, in both left and right upper panels, the lanes 1, 2 and 3 show the WS-protein fractions recovered after first, second and third consecutive washes in buffer A to solubilize WS-proteins, respectively. Lanes 4 and 5 represent the urea soluble-protein fractions recovered during two consecutive washes of WI-protein pellet (containing membranes) with buffer B containing 8 M urea, respectively. Lane 6 represents the 0.1 N NaOH-solubilized proteins from membranes and the lane 7 from both 1- and 6-month old lenses (left and right panels) show the purified lens membrane preparations. Similarly, lanes 7 and 8 from 6-month old lenses (right panel) represent purified membrane preparation. Lane 9 of 6-month old lenses represents the crude lens WS-homogenate. The results show that the green fluorescence representing WTαA in CRYαA_WT_ mice was entirely disappeared on urea solubilization in 1- and 6-month old lenses (lanes 1 to 5 in both left and right panels), whereas it was still present in these lenses until 0.1 N NaOH wash (lane 6 in left and right panels). In contrast, the green fluorescence still existed in lane 6 of membranes from 1- and 6-month-old CRYαA_N101D_ lenses. Together, the results suggest that αA_N101D_ was tightly bound and at the higher levels to lens membrane of CRYαA_N101D_ lenses relative to CRYαA_WT_ lenses.

**Fig. 6:**
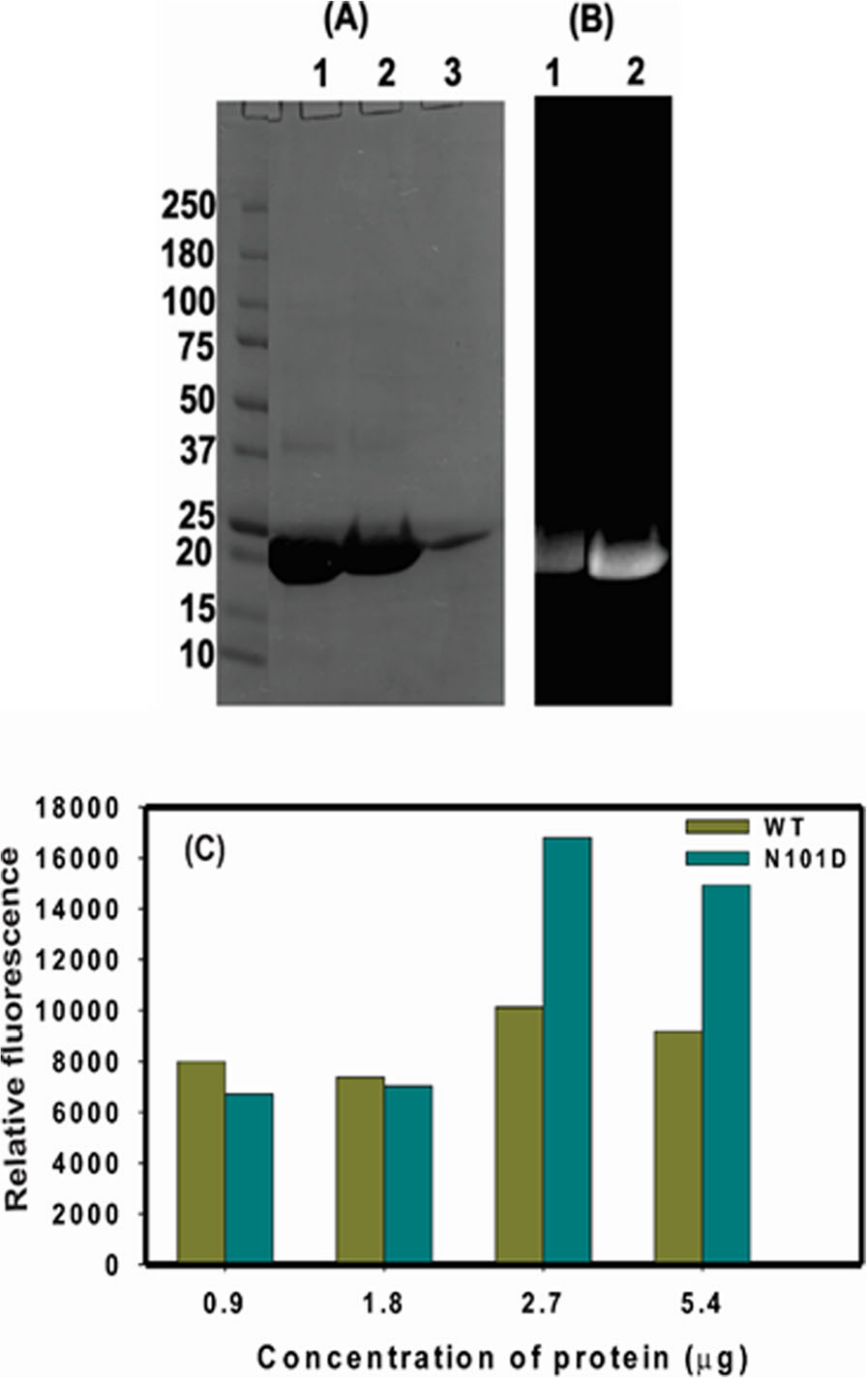
Relative in vitro binding of recombinant αA_N101D_ and WTαA proteins to lens membrane. **a** The recombinant WT αA- and αA-_N101D_ proteins were labeled with Alexa 350, purified by a size-exclusion HPLC column and analyzed by SDS-PAGE. Lane 1: Coomassie blue-stained WTαA, lane 2: αA_N101D_ mutant protein, and lane 3: purified lens membrane from non-transgenic C57 mice. **b** Images of labeled αA_N101D_ and WTαA proteins. Lane 1: Alexa 350-labeled WT αA, and lane 2: αA_N101D_ protein. **c** Binding of a WT αA, and αA_N101D_ with purified lens membrane (2.5 mg protein; isolated from 1- to 3-month old non-transgenic C57 mice). During the binding assay, the protein mixtures were incubated with increasing but identical concentrations of either Alexa-labelled WTαA- or αA-_N101D_ at 37^ο^C for 6 h, centrifuged at 14,000Xg and the supernatant and pellet (membrane fraction) recovered. After washing the membrane fraction with water and centrifugation as above, the relative fluorescence of membranes incubated with WT αA- and αA-_N101D_ mutant proteins was determined. The values reported are the average of triplicate assays

On Image J-quantification of the Western blots (Fig. 5 i and j), the lanes 4 and 5 (urea soluble fractions) of 1-month old lenses showed higher levels (2.5X) of immunoreactivity with anti-His antibody in the CRYαA_N101D_ lenses (shown in red) compared to those from CRYαA_WT_ lenses (blue). Similarly in Fig. 5j, among the lanes 4 and 5 containing same fractions from 6-month old lenses (as described in 1-month old lenses), the lane 5 showed a greater immunoreactive level of CRYαA_N101D_ lenses (red) compared to CRYαA_WT_ lenses (blue). Additionally, the lane 6 (representing membrane remaining after two urea washes, right panel) of 6-month CRYαA_N101D_ lenses exhibited about 2X greater immunoreactivity than CRYαA_WT_ lenses (Quantification results not shown). Together, the results show that relative to CRYαA_WT_, higher levels of CRYαA_N101D_ were tightly associated with the lens membranes of 1- and 6- month old CRYαA_N101D_ mice.

(iii). **Relative Membrane-Binding of Alexa 350-Labeled Recombinant WT**α**A- and** α**A-**_**N101D**_ **Crystallins**
  To examine whether αA_N101D_ show a greater binding affinity to the lens membrane relative to WTαA-crystallin, the binding of the two recombinant proteins to purified lens membrane was determined. The recombinant WTαA- and αAN101D proteins were labeled with Alexa 350 using a protein labeling kit by the procedure described by the manufacturer (Molecular Probes, Thermo fisher Scientific). The two labeled-proteins were purified by a size-exclusion HPLC column and were analyzed by SDS-PAGE. Figure 6a shows the Coomassie blue-stained WT αA (lane 1), αA_N101D_ protein (lane 2), and the purified lens membrane from non-transgenic C57 mice (lane 3). The Fig. 6b shows the images of the two Alexa 350-labeled proteins under a UV trans-illuminator [Lane 1: Images of Alexa 350-labeled WTαA, and lane 2: Alexa 350-labeled αAN101D). During the binding assay, the purified lens membrane (containing 2.5 mg protein; isolated from 1 to 3-month old non-transgenic C57BL mice) was incubated with increasing but identical concentrations of either Alexa-labelled WT αA- or αA_N101D_ proteins at 37^ο^C for 6 h (See details in Methods). A relatively higher levels (> 1.5X) of binding of αAN101D protein relative to WTαA protein with membrane preparation was observed (Fig. 6c). The values reported are the average of triplicate assays.
(iv). **Immunogold-Labeling for Relative Localization of αA-WT and αAN101D in Lens Membranes of CRYαA**_**N101D**_ **and CRYαA**_**WT**_ **Mice**

**Fig. 7:**
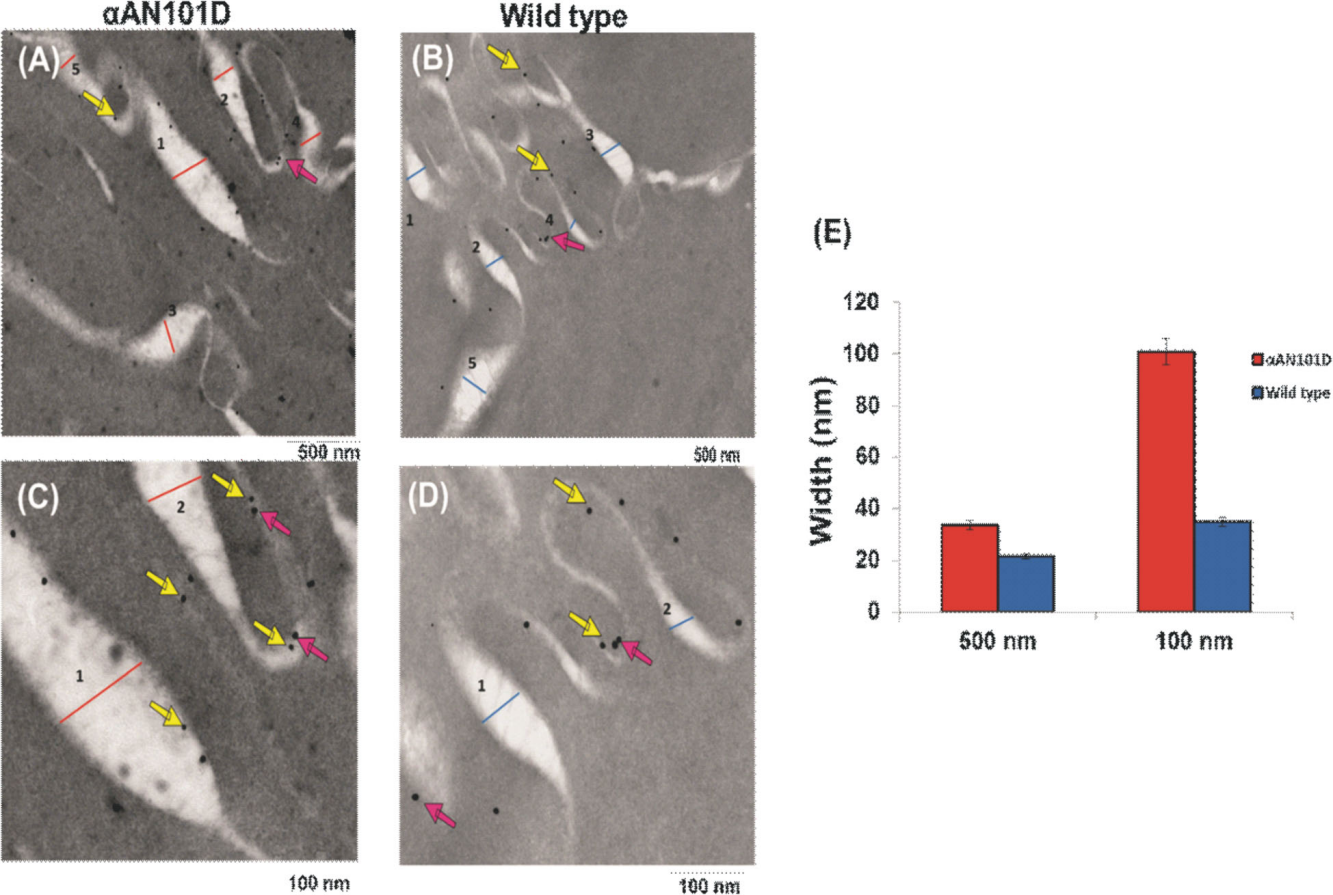
Immunogold-labeling to determine relative localization of αA-WT and αA_N101D_ in lens membranes of CRYαA_N101D_ and CRYαA_WT_ mice. **a** and **c** show membranes of lenses from CRYαA_N101D_ (at 500 nm and 100 nm magnification respectively) and (**b**) and (**d**) from CRYαA_WT_ (at 500 nm and 100 nm magnification respectively). The bigger particles (25 nm, red arrows) represented the aquaporin 0 whereas the smaller gold particles (10 nm, yellow arrows) represented the His-tagged αA_N101D_ and WTαA-. As shown in the representative images in (**a**) to (**d**), both 10 nm and the 25 nm gold particles were bound to membranes.**e**: Quantification of width of membranes from lenses of CRYαA_N101D_ and CRYαA_WT_ mice. Note that the lens membranes of CRYαA_N101D_ mice were about 2X wider that those from CRYαA_N101D_ mice, suggesting membrane swelling of the former lenses

To ascertain the relative levels association αAN101D vs. WTαA to the lens membrane in vivo, the immunogold-labeling experiment was carried out (See details in Methods). (A) and (B) in Fig. 7 show lens membranes from CRYαA_N101D_ and CRYαA_WT_ mice at 500 nm magnification, and (C) and (D) from these lenses at 100 nm magnification, respectively. The bigger gold particles (25 nm, red arrows) the smaller gold particles (10 nm, yellow arrows) represented the aquaporin-0 and the His-tagged αAN101D and WTαA, respectively. As shown in the representative images in (A) to (D), the 25 nm gold particles (representing aquaporin-0, identified by red arrows) were bound to membranes. On counting the membrane-associated 10 nm particles (representing His-tagged αAN101D and WTαA), almost the same numbers of the particle were found to be associated with membranes of both CRYαA_N101D_ and CRYαA_WT_ lenses, suggesting that the His-tagged αAN101D and WTαA were bound to the membranes of the two types of lenses. Our previous study [28] showed that αAN101D protein constituted about 14 and 14.2% of the total αA- crystallin in the WS- and WI-proteins, respectively in the lenses of CRYαA_N101D_ mice. Therefore, an argument can be made that although an almost equal number of 10 nm and 25 nm particles were associated with membranes of the two type of lenses, a higher number of gold particle representing αAN101D relative to WTαA were associated with the membrane.

Another interesting observation was that the membranes of CRYαA_N101D_ lenses were about 2X more swollen relative to those of CRYαA_WT_ lenses [Fig. 7, compare (A) to (B) and (C) to (D)]. The width of the membrane was quantified using Image J as shown in Fig. 7e. The swelling could represent water intake within the lens cells due to the potential ionic imbalance in the CRYαA_N101D_ lenses compared to CRYαA_WT_ lenses. Such a possibility of ionic imbalance was further determined as described below.

### Na, K-ATPase and Ca^2+^ levels in cultured epithelial cells from lenses of CRYαA_N101D_ and CRYαA_WT_ mice

**Fig. 8:**
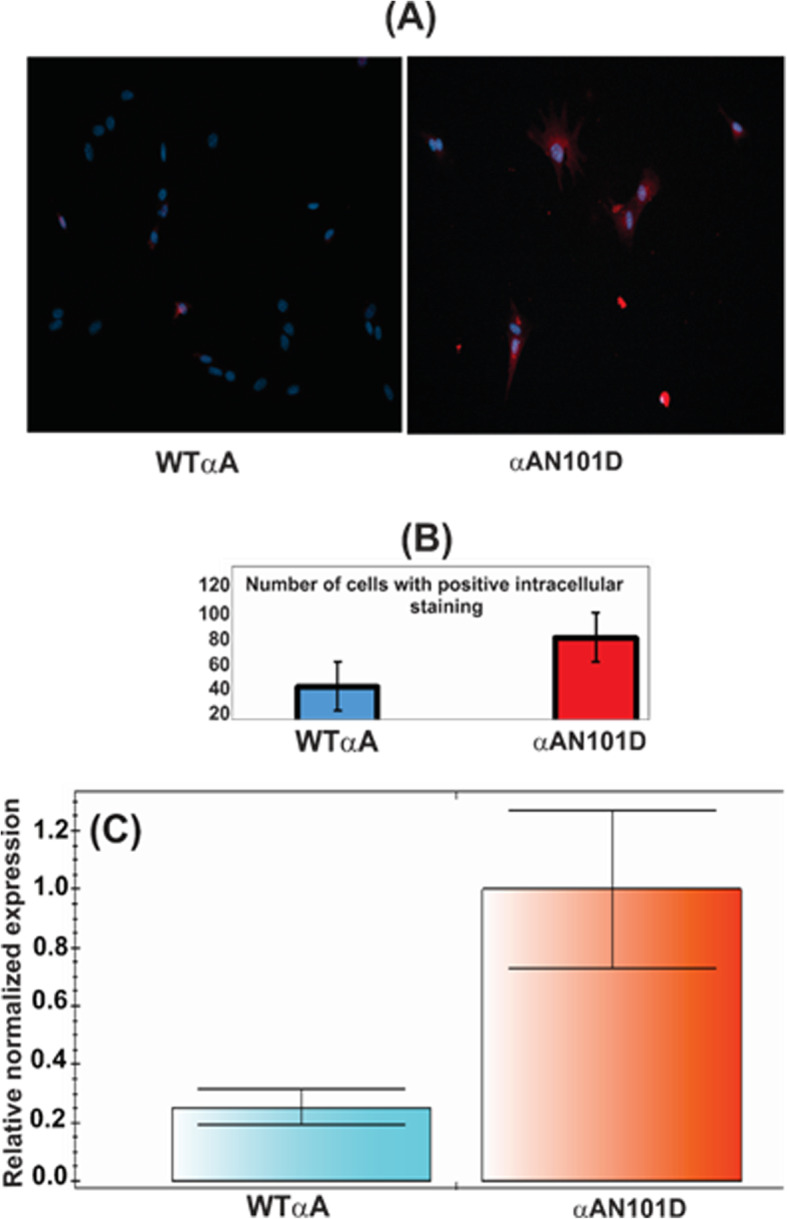
Determination of levels of intracellular Ca^2+^ and Na, K-ATPase mRNA in cultured epithelial cells from lenses of CRYαA_N101D_ and CRYαA_WT_ mice. **a** Left and right panels show intracellular Ca^2+^ staining following uptake from calcium orange in cells from CRYαA_WT_- and CRYαA_N101D_ mice, respectively. **b** Quantification by Image J of the number of cells that showed positive intracellular Ca^2+^-staining following uptake from calcium orange in CRYαA_WT_ - and CRYαA_N101D_ cells. **c** Relative levels of Na, K-ATPase mRNA in epithelial cells from CRYαA_WT_- and CRYαA_N101D_ mice as determined by the QRT-PCR method

Sodium-potassium-adenosine triphosphatase (Na, K-ATPase) has been recognized for its role in regulating electrolyte concentrations in the lens, and the electrolyte balance is vital to lens transparency [35, 36]. In addition, calcium has been reported to control both sodium and potassium permeability through lens membranes [37]. In our previous study [29], we showed that the expression of Na,K-ATPase at the protein level was drastically reduced in CRYαA_N101D_ lenses relative to CRYαA_WT_ lenses. Next, the levels of Na, K-ATPase mRNA, and Ca^2+^ levels were determined in cultured epithelial cells from lenses of CRYαA_N101D_ and CRYαA_WT_ mice. Both (A) and (B) in Fig. 8 show intracellular Ca^2+^ levels in the presence of calcium orange in cultured epithelial cells from CRYαA_N101D_ and CRYαA_WT_, respectively. Only a few CRYαA_N101D_ epithelial cells showed the Ca^2+^ uptake, which was possibly due to our previous finding that the lens cells contained only about 14% of αAN101D mutant protein [28]. In this experiment, 100 cells from the cultures of two types of lenses were counted. On quantification by Image J, the number of cells exhibiting calcium orange uptake were 1.5X greater in cells of CRYαA_N101D_ lenses relative to cells from CRYαA_WT_ lenses (Fig. 8b). On the determination of levels of mRNA of Na, K-ATPase in these cells, its level was 75% lower in the CRYαA_N101D_ lens cells than CRYαA_WT_ lens cells (Fig. 8c).

## Discussion

Several past studies have shown in vitro effects of deamidation of crystallins on their structural properties including those in αA-, and αB-crystallins [21–25]. It has been reported that deamidation of Asn and Gln was the major modification identified in several human cataractous and aged lenses and these totaled 66% of the modification in the water-soluble and water-insoluble protein fractions that was analyzed by 2D LC/MS [23]. The mass spectrometric analysis found that there is negligible (less than 1%) deamidation at αA_N101_ site in both aged and cataractous human lenses [38]. These studies suggested that because of low levels of deamidation of αA- at N101 to D in normal and cataractous lenses, the αAN101D might not play a significant role in cataract development. However, additional studies suggest otherwise. For example, our in vitro studies showed significant altered structural and functional properties of αA-crystallin on deamidation of N101 residue but not of N123 residue [24]. We also showed that the WS-protein fraction from 50 to 70 year-old human donors contained αA- fragments with deamidation of N101 to D [39]. This finding is significant because recent studies have also shown an increasing role of crystallin fragments in cataract development [40, 41]. In the present study, the cortical cataract development in mice on the introduction of αA-N101D transgene further show significance of deamidation of this site and altered changes in the lens. However, the exact in vivo molecular mechanism of αAN101D-induced crystallin’s aggregation is yet to be fully understood.

Previously we showed that the three recombinant deamidated αA-mutants (N101D, N123D, and N101D/N123D) exhibited reduced levels of chaperone activity, alterations in secondary and tertiary structures, and larger aggregates relative to WT-αA-crystallin [24, 25]. Among the above three mutants, the maximally affected and altered properties were observed in the recombinant αAN101D mutant [25]. Additionally, our recent results show that in vitro*,* the deamidated αA-, and αB-crystallins facilitated greater interaction with βA3-crystallin, leading to the formation of larger aggregates, which might contribute to the lens cataractogenic mechanism [42]. As a further extension of our previous studies [28, 29], in some studies the 7-month old lenses were chosen because of the development of cortical cataract at about 7-month of age in the αA-_N101D_ mice relative to αA_wt_ mice. In other experiments, lenses from 5-month old of both types of mice were used to determine the progression of phenotypic changes in lenses to determine their significance in cataractogenic mechanism.

The present study show that the introduction of αAN101D trans-gene in a mouse model resulted in the following major in vivo effects in lenses of CRYαA_N101D_- relative to CRYαA_WT_ mice: (A) An age-related difference in protein profiles with an increasing association αA_N101D_ with WI-protein fraction suggesting its insolubilization after 4-months of age. (B) The WS-HMW protein fraction showed a higher level of proteins with a greater M_r_. (C) Mass spectrometric analysis showed preferential insolubilization of αA-, αB-, γD- and γE-crystallins, and nestin, which remained insoluble even in 8 M urea. (D) The tight association of αAN101D with membranes relative to WTαA, which could not be fully dissociated with 8 M urea treatment. (E) In vitro, αA_N101_ a showed greater affinity and binding to lens membranes relative WTαA. (F) The greater number of immunogold-labeled αA_N101_ relative WTαA binding to membrane along with relatively greater swelling of lens membranes, suggesting the potential water uptake due to intracellular ionic imbalance, and (G) The ionic imbalance was suggested by the greater Ca^2+^ uptake and 75% reduction in mRNA levels of Na, K-ATPase in the epithelial cells cultured from CRYαA_N101D_ lenses relative to those from CRYαA_WT_ lenses. Our mass spectrometry analysis showed that retinal dehydrogenase was absent in the N101D mice. It has been shown earlier that *Aldh1a1*(−/−) knock-out mice developed lens opacification later in life [43]. Retinal dehydrogenase 1 may protect the lens against cataract formation by detoxifying aldehyde products on lipid peroxidation in both cornea and lens. It has been shown that antimalarial drug chloroquine which binds and inhibit retinal dehydrogenase 1 [44] induce cataract in rats [45]. Together, these findings suggest altered membrane integrity (possibly due to greater levels of αA_N1010D_ binding to membrane than WTαA) resulting in intracellular ionic imbalance in CRYαA_N101D_ lenses, which could play a major role in the cortical cataract development.

Among the lens crystallins, only α-crystallin show an association with the membrane in both normal and cataractous lenses [6, 46–50]. Lens membranes contain both a high-affinity saturable and low-affinity non-saturable α-crystallin-binding sites [46, 50–52]. Alpha-crystallin binding to native membranes was enhanced on stripping of extrinsic proteins from the lens membrane surface to expose lipid moieties [32, 33], which contradicted a previous report that the crystallin mostly interacts with lens membrane MP26 protein [53]. Even after stripping extrinsic membrane proteins by alkali-urea treatment, the full-length αA-, and αB-crystallins remained associated with membranes of both bovine and human lenses [6]. Additionally, αB-crystallin showed three-fold higher binding to lens membrane relative to αA-crystallin, and their binding was affected by the residual membrane-associated proteins, suggesting that their binding behaviors were affected by an intrinsic lens peptides [6]. A large-scale association of proteins with cell membranes in the lens nucleus (mostly in the barrier region) occurs after middle age in human lenses [48], and such association was enhanced by mild thermal stress [49]. The in vitro studies further supported this because the binding capacity of α-crystallin from older lenses to lipids increased with age and decreased in diabetic donors who were treated with insulin [50]. This implied that under diabetic conditions, abnormal binding of α-crystallin to lens membrane occurred. Such information in the literature about membrane binding of native vs. post-translationally modified crystallins including the deamidated αAN101D species is presently lacking. Therefore, the results of the present study showing relatively increased binding of αAN101D relative to WTαA are highly significant.

The RNA sequence and IPA data of our previous study [29] further support the findings of the present study. This study [29] showed that the genes belonging to gene expression, cellular assembly, and organization, and cell cycle and apoptosis networks were altered, and specifically, the tight junction-signaling and Rho A signaling were among the top three canonical pathways that were affected in the CRYαA_N101D_ lenses relative to CRYαA_WT_ lenses. The present study showed an increased association of αAN101D to membrane, and this could lead to potential ionic imbalance affecting tight junction assembly and RhoA GTPase expression. This in turn causes increased proliferation and decreased of differentiation and denucleation of epithelial cells, and an accumulation of nuclei and nuclear debris in the lens anterior inner cortex and fiber cell degeneration. Some of these phenotypic changes could be cause or effects, but together could be responsible for the age-related cortical cataract development in CRYαA_N101D_ lenses.

To maintain ionic balance within lens cells, a permeability barrier close to the surface of the lens is responsible for the continuous sodium extrusion via Na, K-ATPase-mediated active transport [35–37]. Without an active sodium extrusion, lens sodium and calcium contents are shown to increase resulting in lens swelling that leads to loss of lens transparency [35]. Similarly, an excessive intracellular Ca^2+^ levels can be detrimental to lens cells, and its increased levels play an important role in development of cortical cataract [37]. Therefore, homeostasis of Na^+^, K^+,^ Ca^2+^ and other ions within the lens has been recognized as of fundamental importance in lens pathophysiology. These have been altered as shown in our present and our previous studies [29]. It is also possible that the increased Ca^2+^ levels could in turn lead to calpain activation and proteolysis of crystallins, which will be investigated in future.

Similar to our study, other studies have shown that an increased membrane binding of α- crystallin in the pathogenesis of different forms of cataracts. The high molecular weight complexes (HMWCs), comprised of α-crystallin and other crystallins, accumulate with aging and show a greater membrane binding capacity than native α-crystallin [50]. Other mutants of αA-crystallin, like the αA_N101D_ mutant, also exhibit a greater membrane binding than corresponding wild-type species [54]. For example, in the αAR116C-associated congenital cataracts, an increased membrane binding capacity along with changes in complex polydispersity, and the reduction of subunit exchange were considered potential factors in the cataract pathogenesis [54]. Similarly, αA-crystallin R49C neo mutation influenced the architecture of lens fiber cell membranes and caused posterior and nuclear cataracts in mice [55].

Interactions between proteins and the cell membrane are an integral aspect of many biological processes, which are influenced by compositions of both membrane lipids and protein structure [56]. Reports have shown the age-related lipid compositional changes in the lens membrane, which might affect α-crystallin binding, i.e., in the nucleus of the human lenses, the levels of glycerophospholipids declined steadily by age 40 as opposed to the levels of ceramides and dihydroceramides increased approximately 100 fold during middle-age [57, 58]. Further, it has been shown that because of the elevation of sphingolipid levels with species, age, and cataract, lipid hydrocarbon chain order, or stiffness increased. Therefore, the increased membrane stiffness caused an increase in light-scattering, reduced calcium pump activity, altered protein-lipid interactions, and perhaps slow fiber cell elongation [60]. Presently, whether similar changes occur in αA_N101D_ lenses are not known.

Alpha A- and αB-crystallins differently associate with the cellular membrane, i.e. αA-crystallin may interact exclusively with membrane phospholipids, and thereby unaffected by the presence of extrinsic proteins on the membrane, whereas these proteins may act as conduits for αB-crystallin to bind to the membrane [58]. Presently, the specific binding mechanism of αAN101D to the membrane and age-related changes in lipid composition in lenses CRYαA_N101D_ vs. CRYαA_WT_ are unknown, and these are presently the focus of our investigations.

## Conclusions

The results presented in this study suggest that an increased association of αAN101D relative WTαA with the lens membrane causes a possible loss of membrane integrity, leading to an ionic imbalance, and in turn, to membrane swelling, cellular disorganization and finally cortical opacity. Our future study will determine the specific binding site in the αAN101D relative WTαA, and changes in the membrane compositions that might facilitate the increased binding of the deamidated crystallin with the membrane.

## Supplementary Information

**Additional file 1:** **Supplementary Table A.** Water Insoluble-Urea Soluble (WI-US)-Protein Fraction of Alpha A-WT lenses.

**Additional file 2:** **Supplementary Table B.** Water Insoluble-Urea Insoluble (WI-UI)-Protein Fraction of AlphaA N101D lens

**Additional file 3:** **Supplementary Table C.** Water insoluble-urea soluble alphaAN101D (Mr > 30 kDa)

**Additional file 4:** **Supplementary Table D.** Water insoluble-urea insoluble alpha A N101D (Mr > 30 kDa)

**Additional file 5.**

## Data Availability

The datasets analyzed in the current study are available from the corresponding author for reasonable requests.

## Abbreviations

CRYαA_WT_: Wild-type alpha-A crystallins
CRYαA_N101D_: Alpha-A-crystallin in which Asparagine (N) at 101 position was replaced with Aspartic acid (D)
Atp1a2: ATPase Na+/K+ transporting subunit alpha 2
Na, K-ATPase: Sodium-Potassium-Adenosine triphosphatase
HMW: High Molecular Weight
His-tagged: Histidine tagged
WI-US: Water Insoluble-Urea Soluble
WI-UI: Water Insoluble-Urea Insoluble
Aldh1a1: Aldehyde Dehydrogenase 1 Family Member A1
SDS-PAGE: Sodium dodecyl sulfate- polyacrylamide gel electrophoresis
