# Supplementary material for: Increased Association of Deamidated αA-N101D with Lens membrane of transgenic αAN101D vs. wild type αA mice: potential effects on intracellular ionic imbalance and membrane disorganization
Source: BMC Ophthalmol. 2020 Dec 10;20:484. doi: 10.1186/s12886-020-01734-0 (PMC7726915; doi:10.1186/s12886-020-01734-0)
Supplement: Supplementary file 5 — Additional file 5. [file 12886_2020_1734_MOESM5_ESM.docx]

**Supplementary Methods**

**Miscellaneous Methods**

**Mass Spectrometric Analysis**

The mass spectrometric analysis was carried out at the Targeted Metabolomics and Proteomics Laboratory of the University of Alabama at Birmingham.

**(A) In-Gel Digestion, Nano cHiPLC-Tandem Mass Spectrometry, and Protein Pilot 4.5 Search Queries**

Following SDS-PAGE analysis, the desired gel bands were excised, and an overnight wash removed the excess stain with 50% of 100 mM ammonium bicarbonate/50% acetonitrile. Next, the disulfide bonds were reduced by 25 mM dithiothreitol at 50^o^C for 30 min, and the alkylation of the free thiols groups was carried out with 55 mM iodoacetamide for 30 min in the dark. The excess alkylating agent was removed and the gel pieces were washed twice with a 100 mM ammonium bicarbonate for 30 min, and was evaporated to dryness in a SpeedVac (Savant, Thermo Fisher Scientific, Atlanta, GA) before the addition of 12.5 ng/μl of trypsin (Promega Gold Mass Spectrometry Grade, Madison, WI), and incubated overnight at 37oC. Peptides were extracted twice for 15 min from the gel pieces using a 1:1 mixture of 1% formic acid and acetonitrile, then the extracts were and evaporated to dryness, and the samples were resuspended in 30 μl of a 0.1% formic acid before mass spectrometric analysis.

An aliquot (5 μL) of each digest was loaded onto a Nano cHiPLC 200 μm x 0.5 mm ChromXP C18-CL 3 μm 120Å reverse-phase trap cartridge (Eksigent, Dublin, CA) at 2 μL/min using an Eksigent autosampler (Eksigent, Dublin, CA). After washing the cartridge for 4 min with 0.1% formic acid in ddH20, the bound peptides were flushed onto a Nano cHiPLC column [200 μm x 15 cm. ChromXP C18-CL 3 μm 120Å] with a 45 min linear (5-50%) acetonitrile gradient in 0.1% formic acid at 1000 μl/min using an Eksigent Nano1D+LC (Eksigent, Dublin, CA). The column was washed with 90% acetonitrile-0.1% formic acid for 10 min and then re-equilibrated with 5% acetonitrile-0.1% formic acid for 10 min. The SCIEX 5600 Triple-TOF mass spectrometer (AB-Sciex, Toronto, Canada) was used to analyze the protein digest. The IonSpray voltage was 2300 V, and the declustering potential was 80 V. Ion spray, and curtain gases were set at 10 psi and 25 psi, respectively, and the interface heater temperature was 120^o^C. Eluted peptides were subjected to a time-of-flight survey scan from 400-1250 m/z to determine the top twenty most intense ions for MS/MS analysis. Product ion time-of-flight scans at 50 msec were carried out to obtain the tandem mass spectra of the selected parent ions over the range from *m/z* 100-1500. Spectra are centroided and de-isotoped by Analyst software, version TF (Applied Biosystems). A β-galactosidase-trypsin digest was used to confirm the mass accuracy of the mass spectrometer. The tandem mass spectrometry data were processed to provide protein identifications using an in-house Protein Pilot 4.5 search engine (SCIEX) using the Mus musculus (Mouse) UniProt protein database and using a trypsin digestion parameter. All proteins that had at least one peptide with a confidence score of 95% or higher were considered as potential candidates***.***

**(B) In-Solution Digestion Protocol for Mass Spectrometric Analysis**

All of the reagents used were freshly prepared before their use. A 100-μL aliquot of the protein sample (1 mg) in 100 mM Tris buffer (pH 7.8) containing the 6M urea was mixed with the reducing reagent (DTT) and incubated for 1 h at room temperature. Next, 20 μL of the alkylating reagent (iodoacetamide) was added and incubated for 1 h at room temperature, and then 20 *μ*L of the reducing agent was added to consume any unreacted iodoacetamide and allowed to stand at room temperature for 1 h. The urea concentration was reduced to *~*0.6 M by diluting the reaction mixture with 775 μL of water, 100-μL trypsin solution (20 μg of stock trypsin) was added to bring protease-to-substrate ration 1-to-50, and the digestion was carried out overnight at 37°C. The trypsin digestion was stopped by adjusting the pH to <6.0 by adding concentrated acetic acid. The digest was analyzed directly or concentrated by evaporation. As needed, the samples were desalted using a C18 ZipTip™ (Millipore Corporation, Bedford, MA) using manufacturer’s instructions. The mass spectrometric analysis was carried out as described above.
